# Supplementary material for: Adsorption of Peptides onto Carbon Nanotubes Grafted with Poly(ethylene Oxide) Chains: A Molecular Dynamics Simulation Study
Source: Nanomaterials (Basel). 2022 Oct 27;12(21):3795. doi: 10.3390/nano12213795 (PMC9655739; doi:10.3390/nano12213795)
Supplement: Supplementary file 1 [file nanomaterials-12-03795-s001.zip › nanomaterials-1966275-supplementary.pdf]

Supplementary Materials

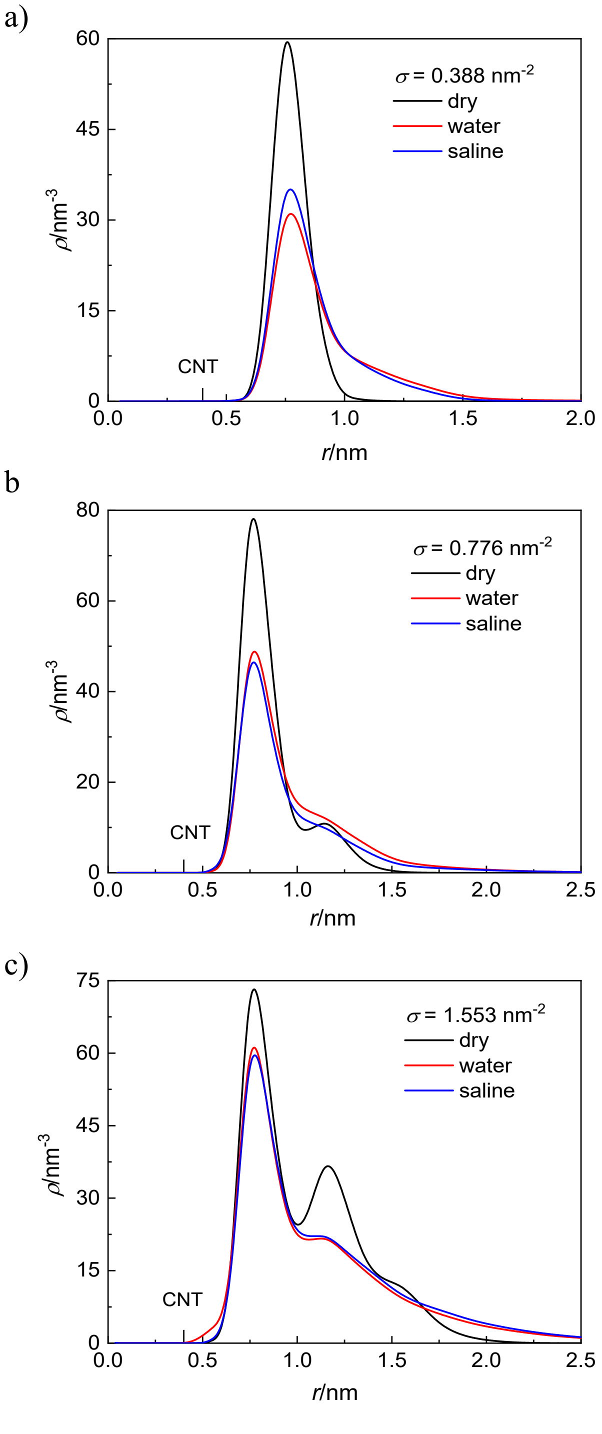

Figure S1. Cylindrical density distribution of the PEO chains grafted at $\sigma = 0.388\ nm^{-2}$ (a), $0.776\ nm^{-2}$ (b), and $1.553\ nm^{-2}$ (c) in the absence of the peptides. The position of the CNT sidewall is also marked.

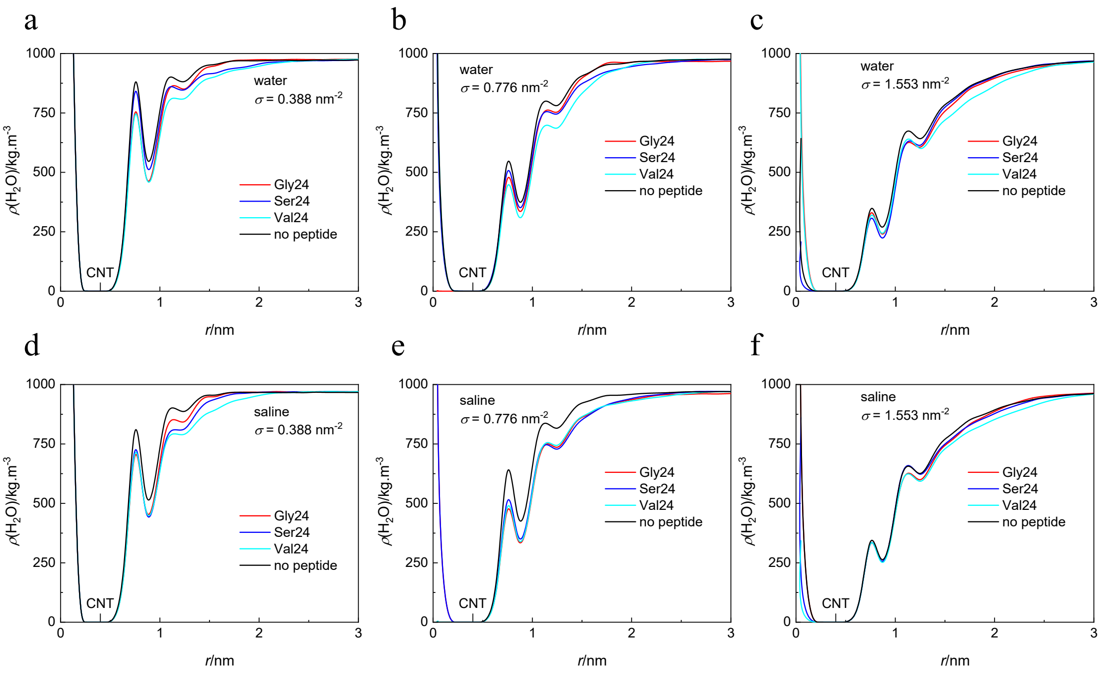

Figure S2. Cylindrical density distribution of water around CNT grafted with the PEO chains at $\sigma = 0.388$ nm^{-2} (a), 0.776 nm^{-2} (b), and 1.553 nm^{-2} (c) in water and at $\sigma = 0.388$ nm^{-2} (d), 0.776 nm^{-2} (e), and 1.553 nm^{-2} (f) in saline in the presence and absence of the peptides.

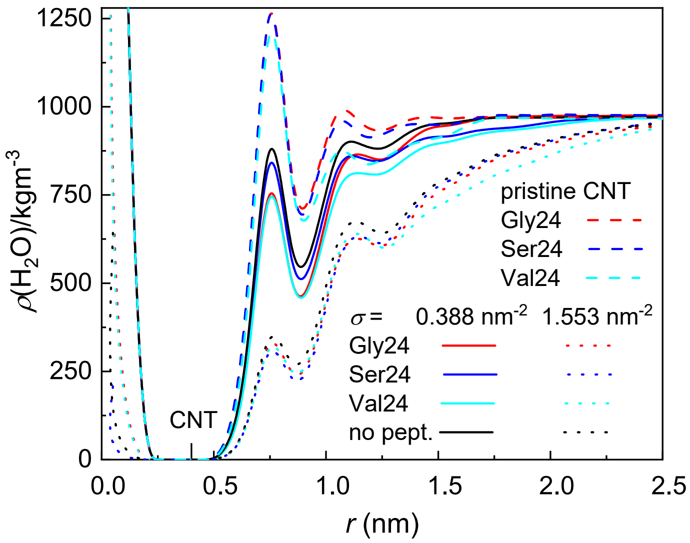

Figure S3. Cylindrical water density distribution in the vicinity of pristine CNT and CNT grafted with the PEO chains at σ = 0.388 nm^{-2} and 1.553 nm^{-2} in the presence and absence of the peptides in water. The position of the CNT sidewall is also marked.

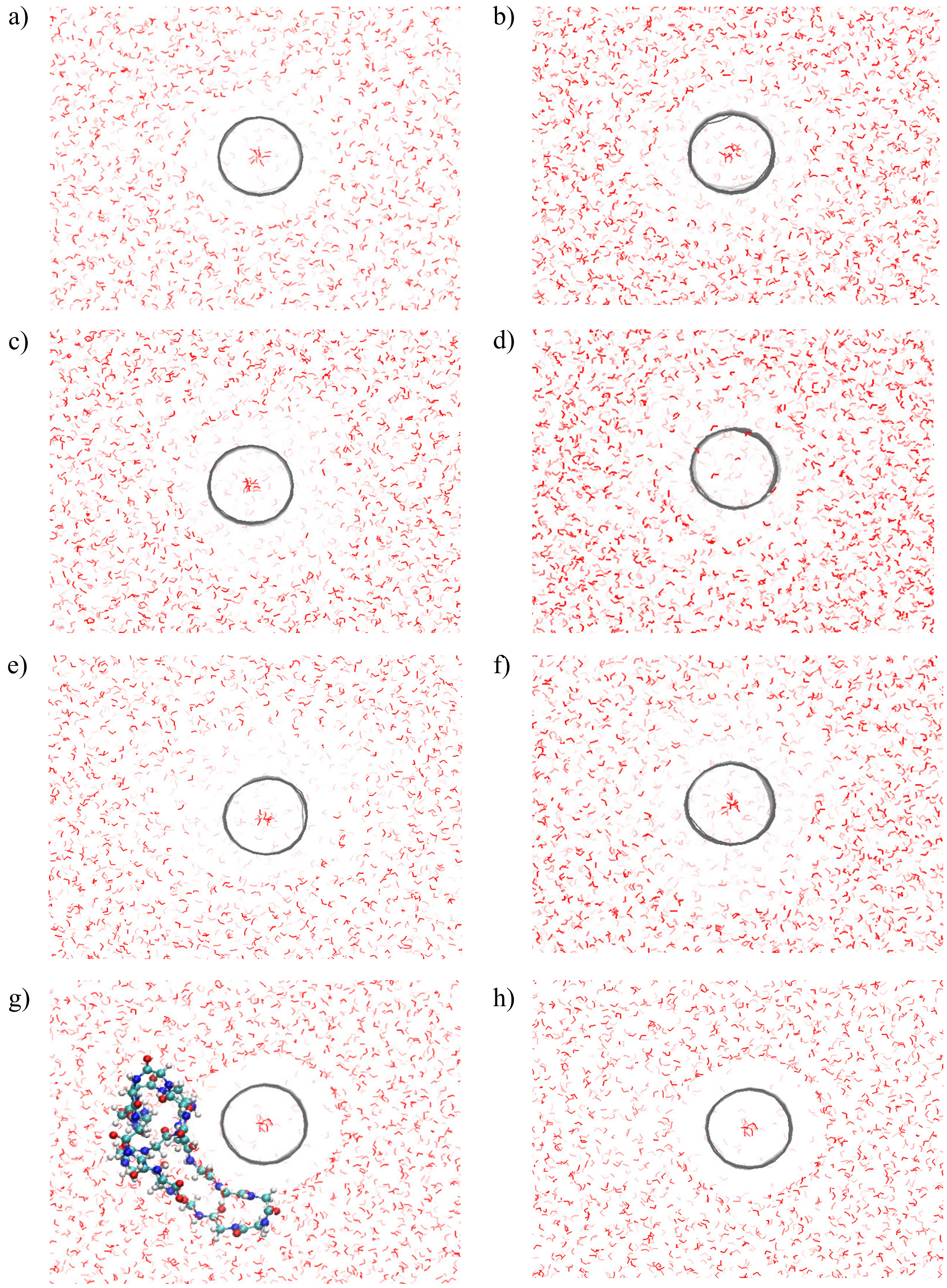

Figure S4. Cross-sectional view of water molecules arranged around CNT grafted with the PEO chains at σ = 0.388 nm^{-2} (a), 0.776 nm^{-2} (c), and 1.553 nm^{-2} (e) in the absence of the peptides and around CNT grafted with the PEO chains at σ = 0.388 nm^{-2} (b),

0.776 nm^{-2} (d), and 1.553 nm^{-2} (f) in the presence of adsorbed polyglycine. The system composed of pristine CNT with adsorbed polyglycine is also presented showing polyglycine (g) and omitting polyglycine (h).

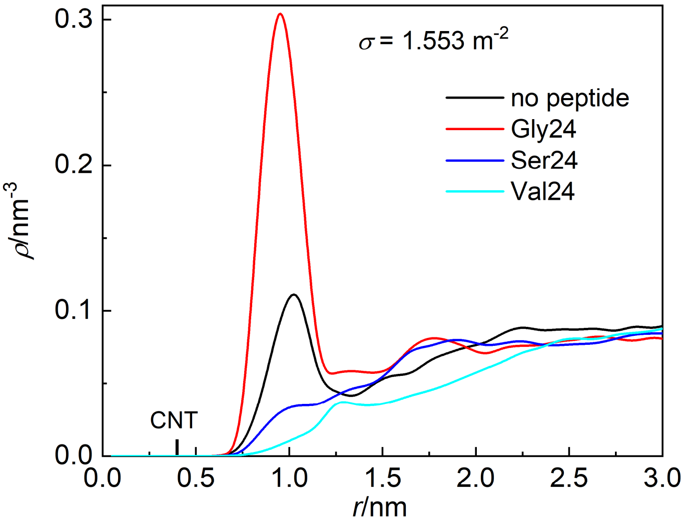

Figure S5. Cylindrical density distribution of sodium cations in the vicinity of CNT grafted with the PEO chains at $\sigma = 1.553$ nm^{-2} in the presence and absence of the peptides. The position of the CNT sidewall is also marked.

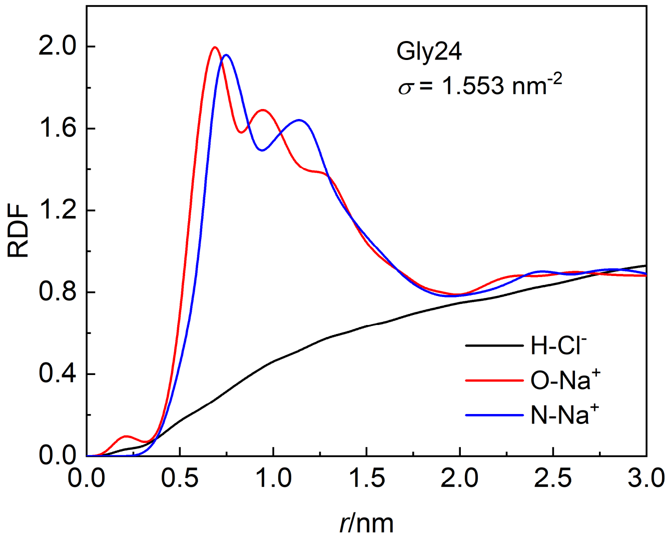

Figure S6. Radial distribution function of sodium cations–polyglycine oxygens, sodium cations–polyglycine nitrogens, and chloride anions–polyglycine amidic hydrogens pairs in system composed of polyglycine and CNT grafted with the PEO chains at σ = 1.553 nm^{-2} in saline.

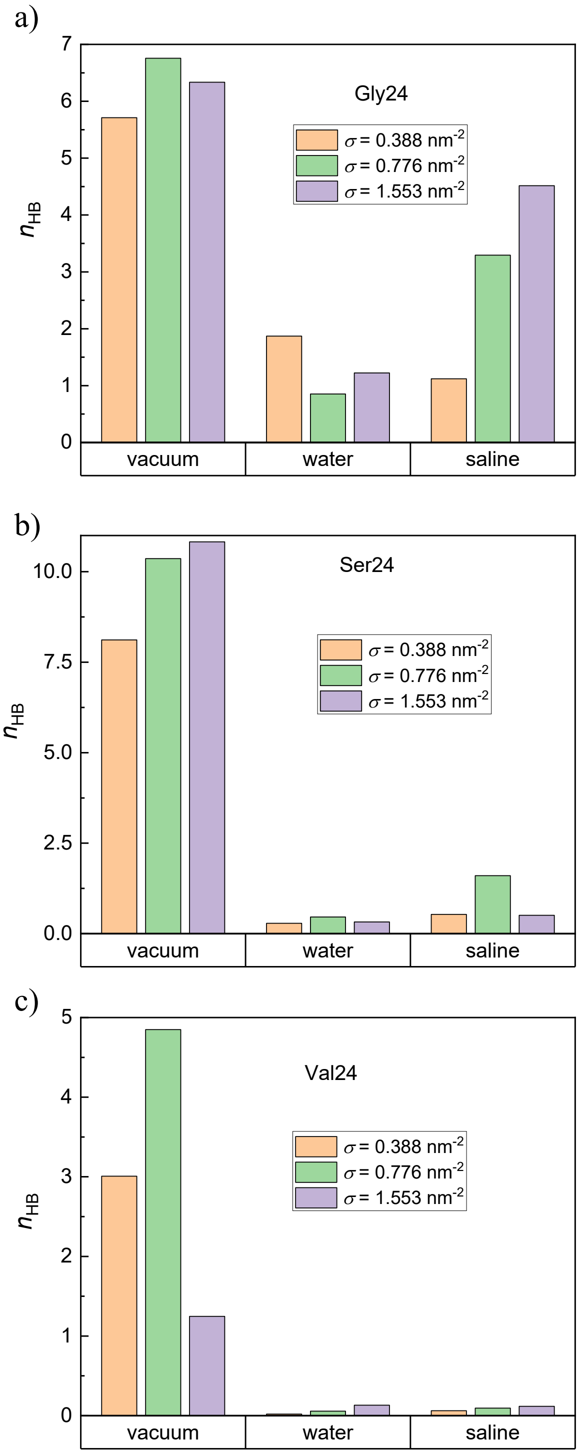

Figure S7. Number of hydrogen bonds formed between the PEO chains with polyglycine (a), polyserine (b), and polyvaline (c) in a vacuum, water, and saline.

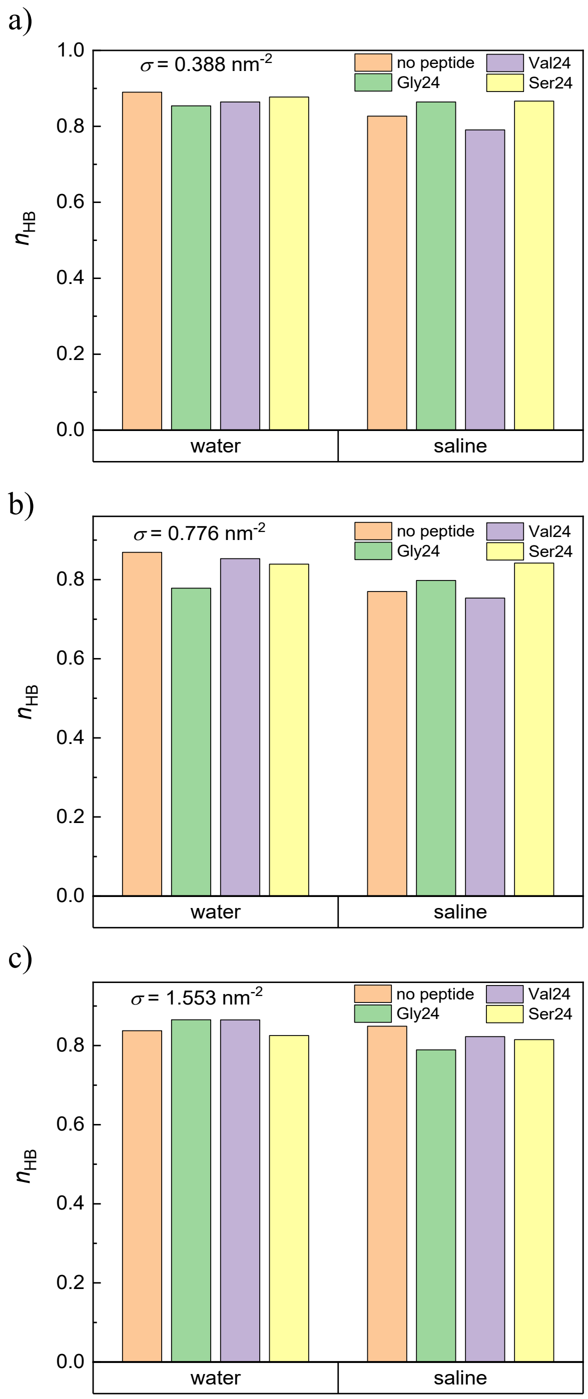

Figure S8. Number of hydrogen bonds formed between water molecules and the PEO chains grafted at σ = 0.388 nm^{-2} (a), 0.776 nm^{-2} (b), and 1.553 nm^{-2} (c) evaluated per one oxygen of the PEO chains in water and saline. For comparison, system without any peptide is also included.

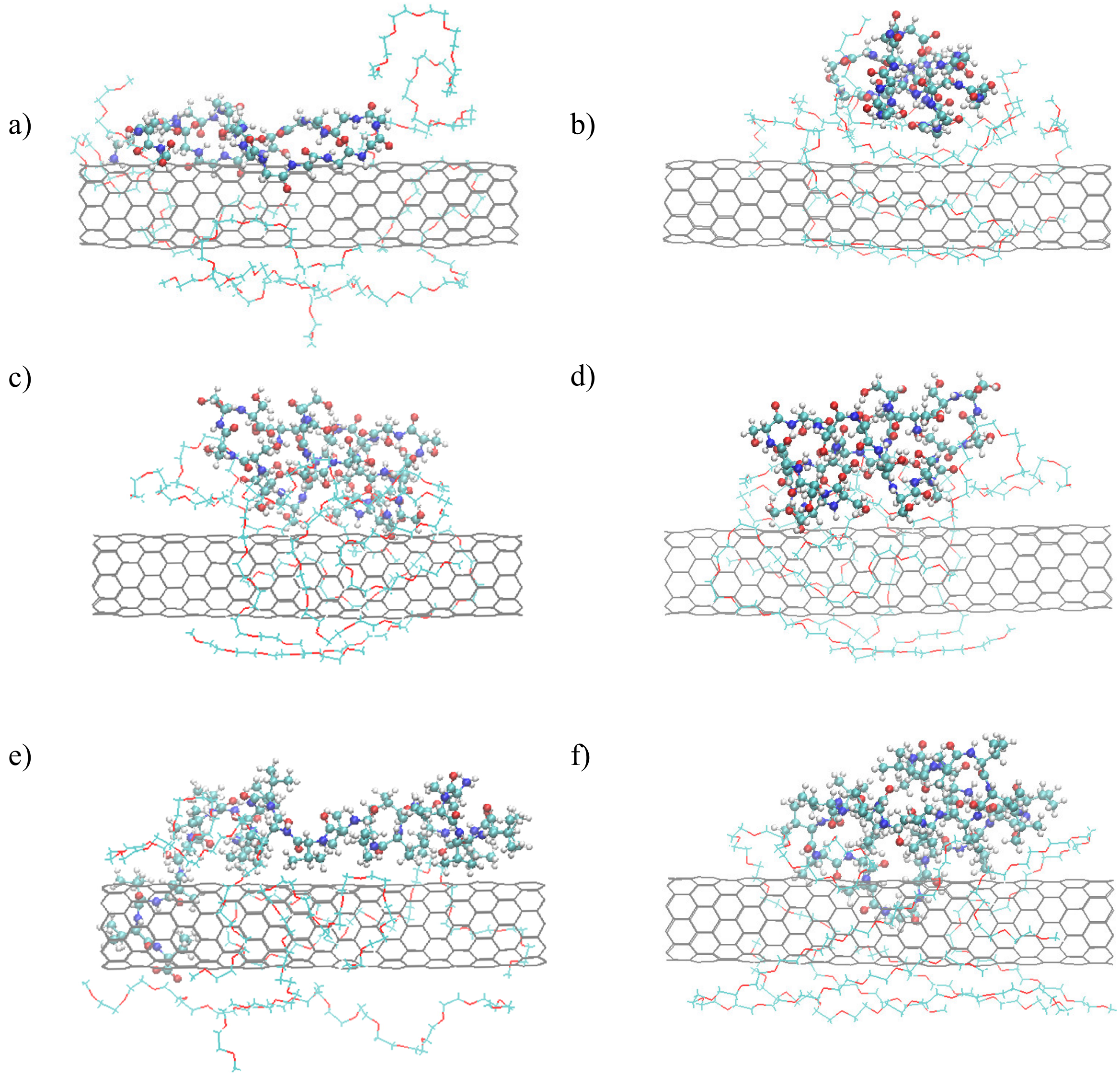

Figure S9. Snapshots of CNT grafted with the PEO chains at σ = 0.388 nm^{-2} interacting with polyglycine (a), polyserine (c), and polyvaline (e) in water and with polyglycine (b), polyserine (d), and polyvaline (f) in a vacuum. Water molecules are omitted for clarity in (a, c, e). The peptides and the PEO chains are represented as balls and sticks and as lines, respectively.

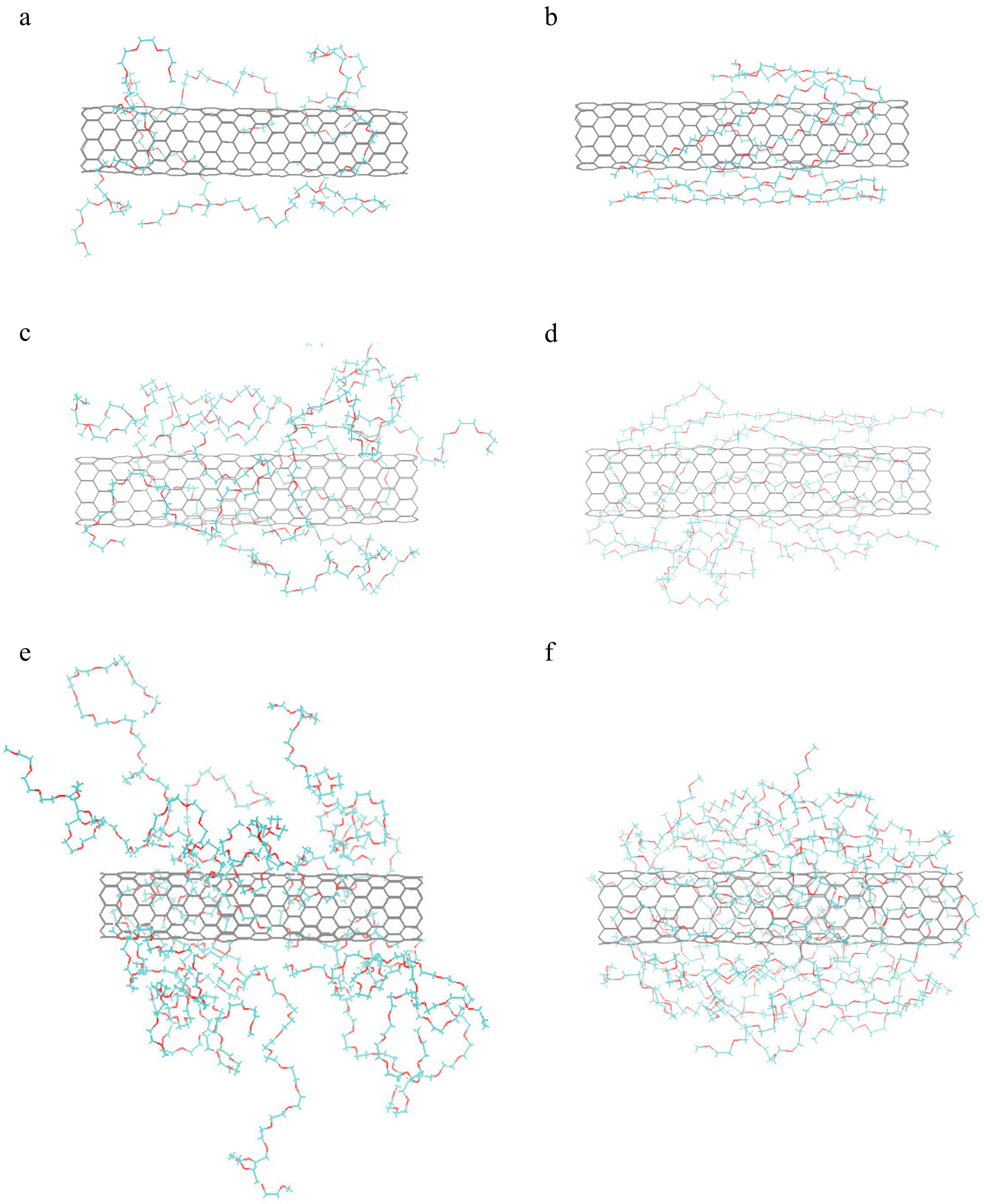

Figure S10. Snapshots of CNT grafted with the PEO chains at $\sigma = 0.388\ \mathrm{nm}^{-2}$ (a), $0.776\ \mathrm{nm}^{-2}$ (c), and $1.553\ \mathrm{nm}^{-2}$ (e) in water and at $\sigma = 0.388\ \mathrm{nm}^{-2}$ (b), $0.776\ \mathrm{nm}^{-2}$ (d), and $1.553\ \mathrm{nm}^{-2}$ in a vacuum (f) in the absence of the peptides. Water molecules are omitted for clarity in (a, c, e).

a)

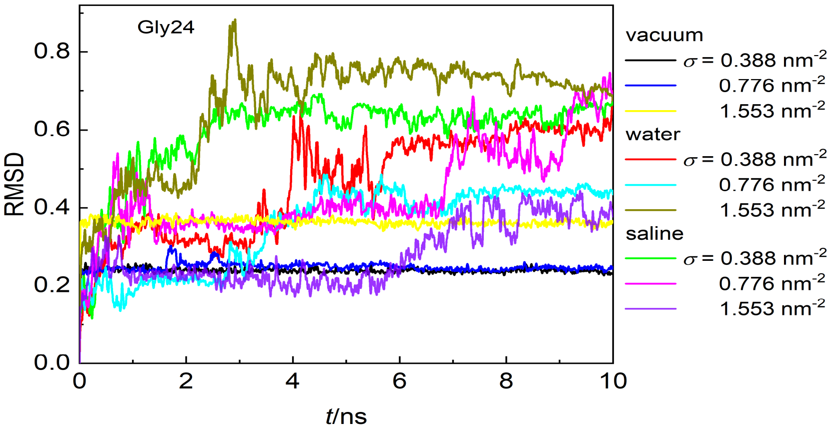

b)

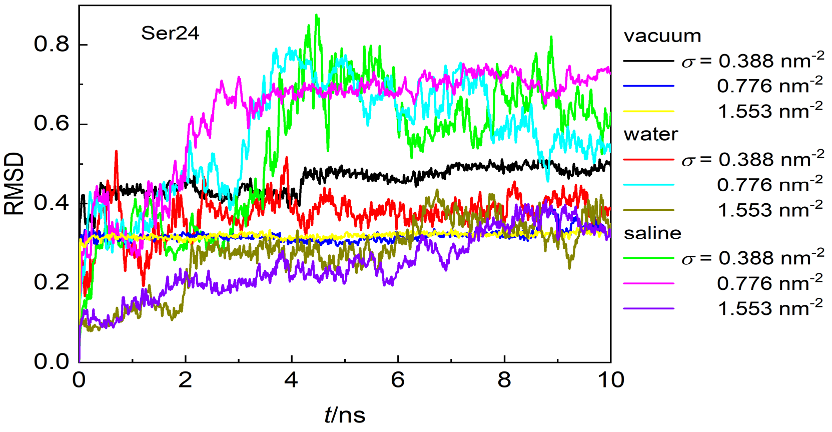

c)

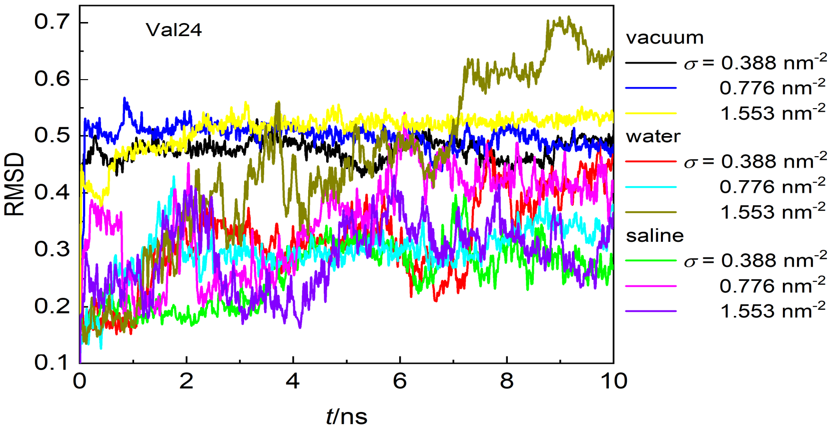

Figure S11. Time evolution of the root-mean-square deviation of polyglycine (a), polyserine (b), and polyvaline (c) interacting with CNT grafted with the PEO chains at different grafting densities, σ, in a vacuum, water, and saline.

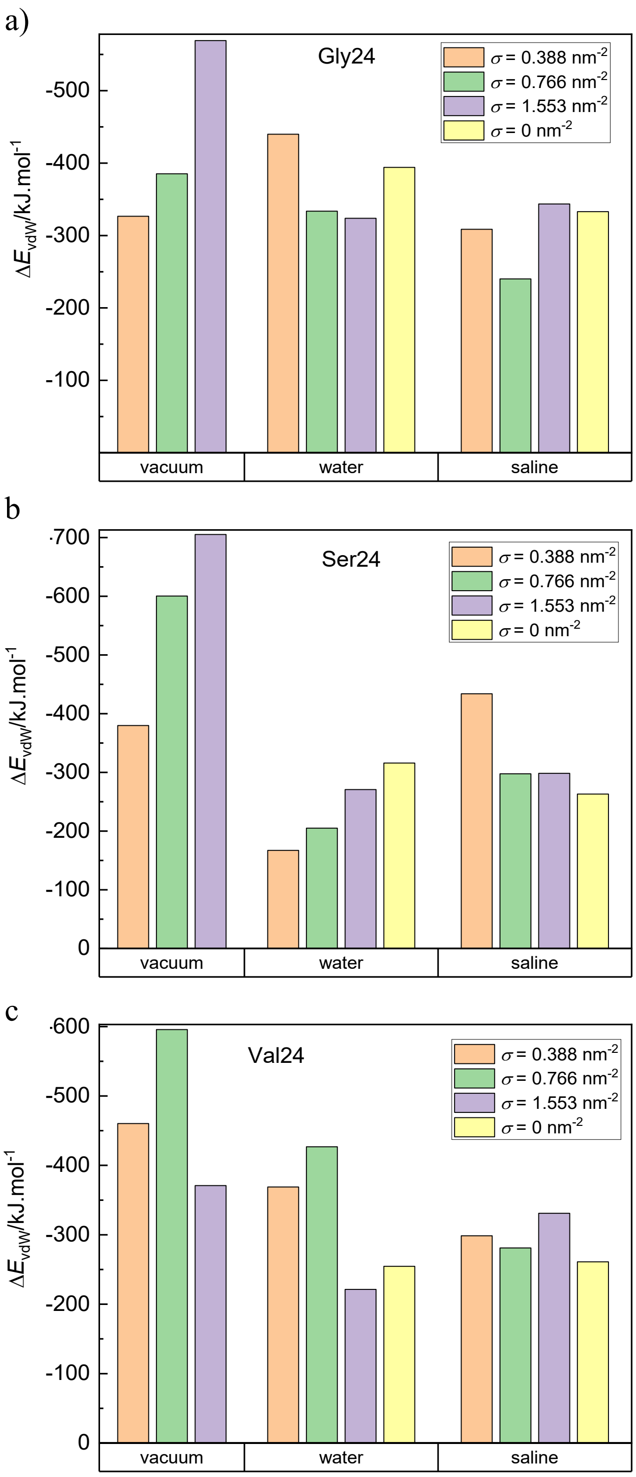

Figure S12. Van der Waals component of the binding free energy of polyglycine (a), polyserine (b), and polyvaline (c) interacting with pristine CNT and CNT grafted with the PEO chains at different grafting densities, σ, in a vacuum, water, and saline.

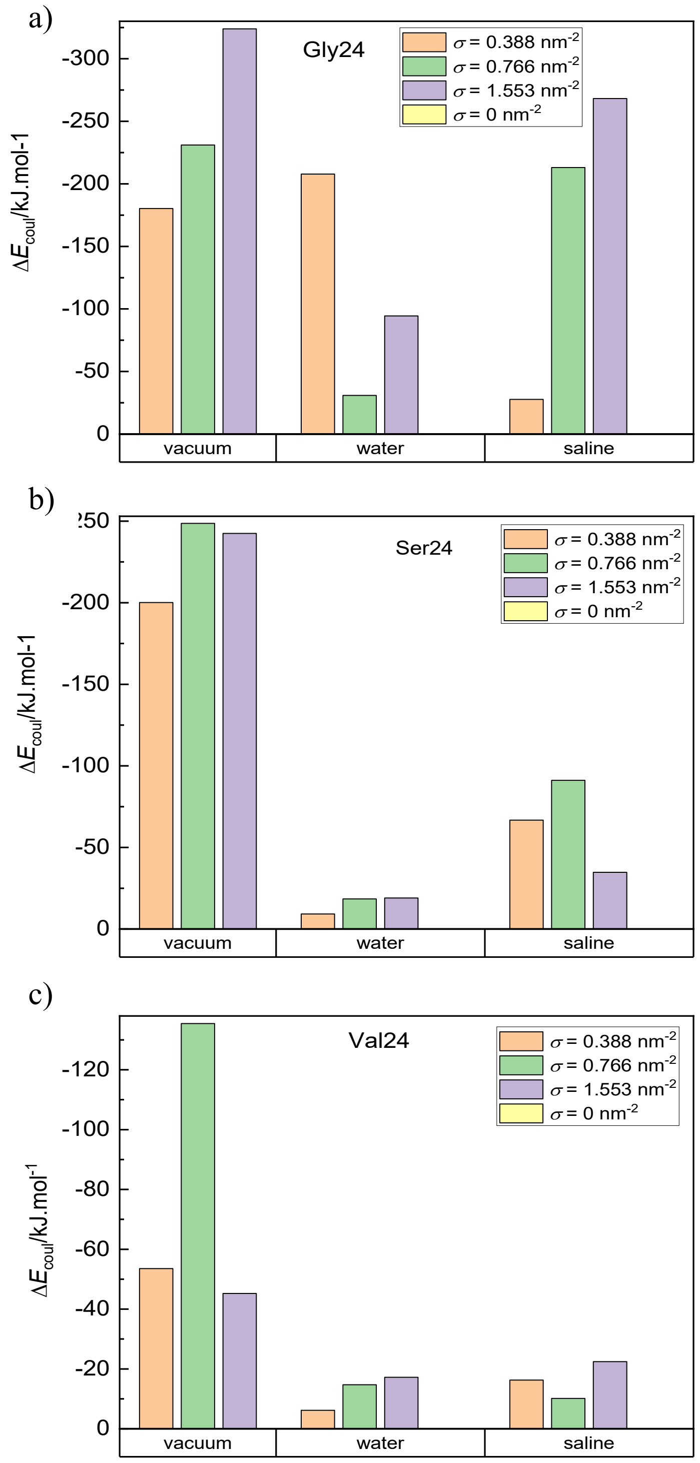

Figure S13. Coulomb component of the binding free energy of polyglycine (a), polyserine (b), and polyvaline (c) interacting with pristine CNT and CNT grafted with the PEO chains at different grafting densities, σ, in a vacuum, water, and saline.

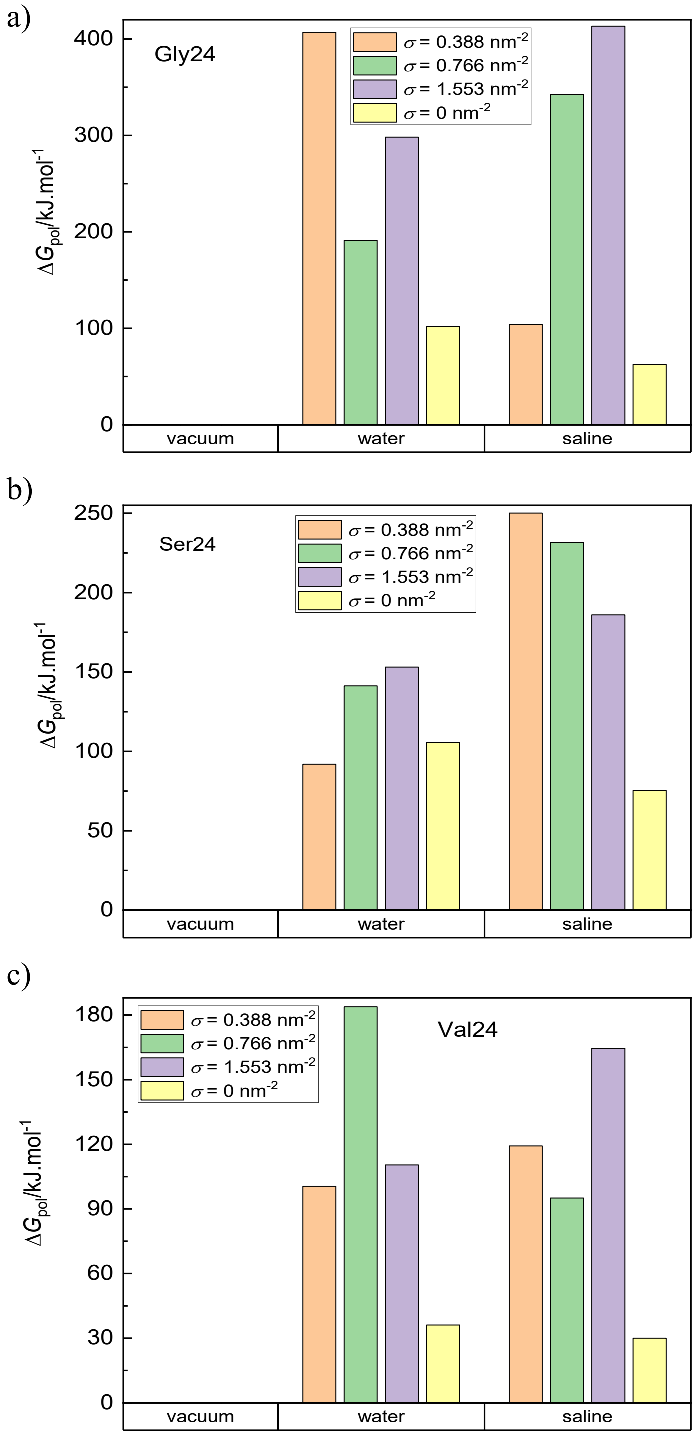

Figure S14. Polar solvation component of the binding free energy of polyglycine (a), polyserine (b), and polyvaline (c) interacting with pristine CNT and CNT grafted with the PEO chains at different grafting densities, *σ*, in a vacuum, water, and saline.

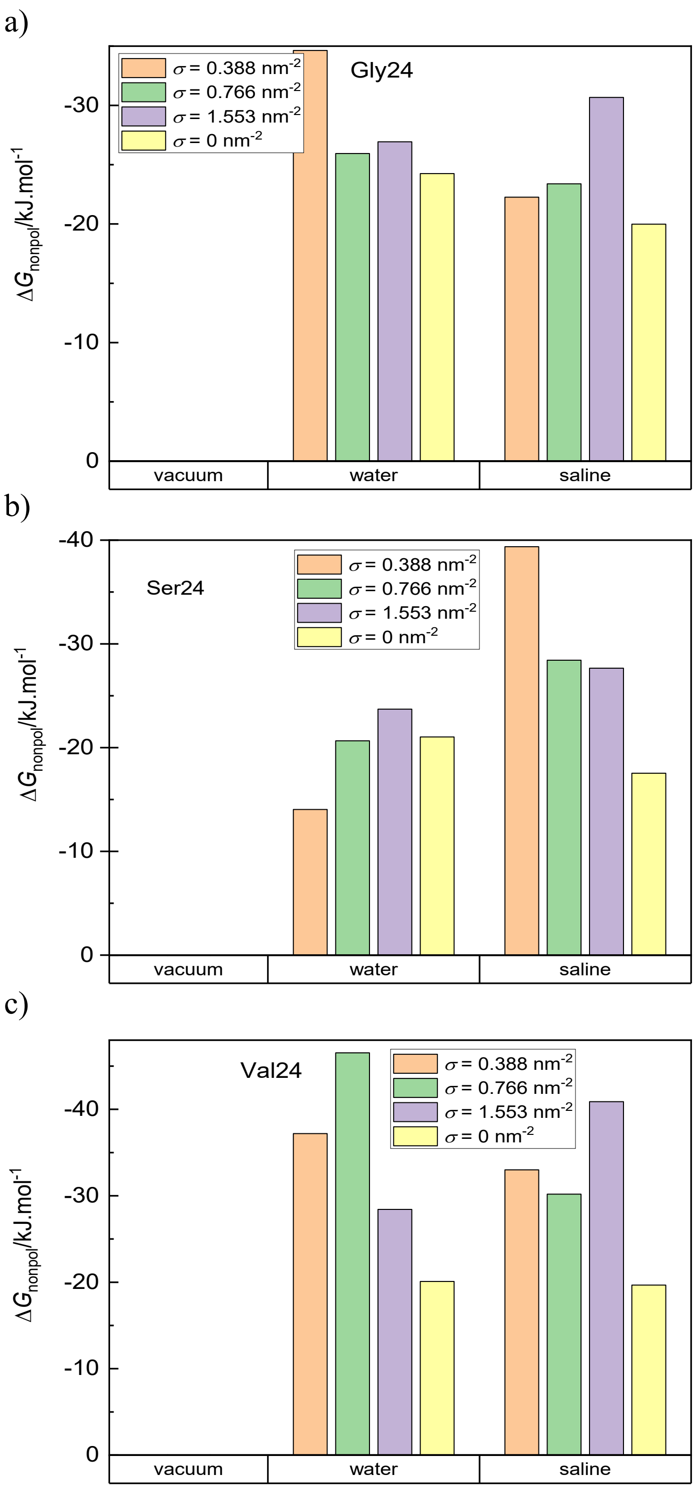

Figure S15. Nonpolar solvation component of the binding free energy of polyglycine (a), polyserine (b), and polyvaline (c) interacting with pristine CNT and CNT grafted with the PEO chains at different grafting densities, σ, in a vacuum, water, and saline.
